# The impact of the initial severity on later outcome: retrospective analysis of a large cohort of botulinum toxin naïve patients with idiopathic cervical dystonia

**DOI:** 10.1007/s00415-020-10128-7

**Published:** 2020-08-05

**Authors:** Harald Hefter, Sara Samadzazeh, Dietmar Rosenthal

**Affiliations:** Department of Neurology, University Hospital of Düsseldorf, Moorenstraße 5, 40225 Düsseldorf, Germany

**Keywords:** Cervical dystonia, Progression of disease, Botulinum neurotoxin, Initial severity, Response behavior

## Abstract

**Background:**

The aim of study was to demonstrate that the first three injections of botulinum neurotoxin type A (BoNT/A) appear to be less effective in botulinum toxin naïve patients with idiopathic cervical dystonia (CD) with mild symptoms and low severity scores (TSUI-scores) at onset of BoNT/A-therapy compared to patients with full-blown CD and high initial TSUI-scores.

**Methods:**

In 337 patients with CD who started BoNT/A-therapy in the BoNT-outpatient clinic of the university hospital in Düsseldorf during the last 12 years, demographical and treatment-related data as well as outcome measures (TSUI-scores) of the first four visits were extracted from the treatment ACCESS data bank.

**Results:**

Distribution of the severity of CD scored using the TSUI-score significantly changed with the first three injections. In patients with a high baseline severity (TSUI-score > 10), mean TSUI-score continuously decreased (*p* < 0.001), whereas in patients with a low initial severity (TSUI-score < 6), mean TSUI-score increased (*p* < 0.001) during the first three injection cycles. Individual responses varied between 100% improvement, no response at all, and even worsening. Improvement of CD at the end of an injection cycle was observed in less than 25% in the mildly affected patients, but in more than 80% in the more severely affected patients.

**Conclusion:**

Clinical response to the first three BoNT/A-injections in severely affected de novo CD-patients is different from the response to BoNT/A in mildly affected de novo CD-patients. This has implications for further scientific studies and the patient management of mildly affected de novo patients with cervical dystonia.

## Introduction

Cervical dystonia (CD) is the most frequent form of idiopathic focal dystonia [1, 2, 3]. The onset is usually insidious, although, in some patients, it may be abrupt. Most patients report deterioration over the initial 5 years, and then symptoms tend to be stabilized [4]. Since the first pilot study in 1985 [5], numerous open as well as double-blind, randomized, placebo-controlled trials have demonstrated and confirmed the efficacy of botulinum toxin type A (BoNT/A) and botulinum toxin type B (BoNT/B) treatment for CD (for an overview, see Ref. [6]).

The clinical effect of a single BoNT-injection usually increases during the first 3 weeks, then maintains a reasonably stable plateau during the next 4–8 weeks, and then declines during the next few weeks [4, 7]. A fairly large percentage of CD-patients (50–90%) responds very well to BoNT-injections (for an overview, see Refs. [4, 6]). Relative improvement (improvement divided by initial severity) was even reported to be similar for all patients irrespective of the severity and complexity of CD [8]. On the other hand, it has been reported that “patients with long-duration dystonia have been found to respond less well than those treated relatively early, possibly because prolonged dystonia produced contractures” [9, 10].

These two observations, which seem to be at variance with each other, were made only a few years after the license of abo-BoNT/A (Dysport^®^; Ipsen) and ona-BoNT/A (Botox^®^; Allergan) in Europe in 1994. Most of the patients treated at that time were botulinum toxin naïve patients with a severe, longer-standing, stable CD responding well to BoNT. The duration from onset of symptoms to onset of therapy could last several years, because only a few specialists offered the new BoNT-therapy. Meanwhile, more than 25 years later, this situation has changed considerably. Numerous movement disorder centers have been founded and intramuscular BoNT-injections have become the first-line therapy for CD [6]. Not only among neurologists but also practitioners CD has become known as a well-treatable neurological disease. Therefore, de novo CD-patients are referred to movement disorder centers earlier than previously, in some cases even a few months after the first clinical manifestation and with mild symptoms only.

Based on the literature that “patients with simple cervical dystonia, such as rotation or tilt, and with shorter duration of symptoms usually respond better” [8] a good clinical outcome after the early onset of BoNT-therapy in these early referred and only mildly affected CD-patients can be expected. However, this has not been analyzed systematically so far.

We, therefore, performed the present retrospective study based on the ACCESS^®^ data bank in our BoNT-outpatient clinic at the university hospital in Düsseldorf (Germany) whether response behavior in de novo CD-patients depends on the initial severity of CD.

## Methods

This monocentric, retrospective study was performed according to the Declaration of Helsinki and the guidelines for GCP. The local ethics committee of the University of Düsseldorf allows publication of anonymized clinical data retrospectively extracted from patients’ charts.

### Retrospective analysis of charts and the BoNT-treatment ACCESS-Data Bank

All patients with idiopathic CD having started their BoNT/A-therapy at the BoNT-outpatient clinic of the university hospital in Düsseldorf (Germany) during the last 12 years were informed on the purpose of this study. Per year, about 25–30 de novo patients with CD are referred to our institution for confirmation of diagnose and initiation of therapy. During the time span of recruitment, patients were seen by only three different physicians who received an official TSUI-score training as members of the Dysport Cervical Dystonia Study group (for details, see Ref. [11]). Patients in whom the BoNT/A-preparation was switched or who had more than one missing injection visit during the first year of treatment were excluded. Nine patients were excluded, because their time intervals between the first three injections exceeded 14 weeks.

From the patients’ charts and corresponding ACCESS^®^ data bank files (*n* = 337), the following data were extracted: (1) demographical data (age at first visit and onset of BoNT-therapy, sex), (2) treatment-related data (dates of the first four injection visits (data of control visits without injection were not taken into account), BoNT/A-preparation used for treatment, and total dose per treatment), and (3) outcome measures (TSUI-scores [12] at these four visits).

For the interpretation of the data, it is important to emphasize that the TSUI-scores were determined at the baseline visit and at week 10–14 after an injection just before the next injection was applied. To compare the results across the use of different BoNT/A-preparations, the following conversion in unified dose units (uDU) was performed: abobotulinumtoxin (Dysport^®^) doses were left unchanged, ona- (Botox^®^) and incobotulinumtoxin (Xeomin^®^) doses were multiplied by 3. These conversion ratios (1:3:3) follow a European consensus recommendation [13].

Part of the recruited patients (*n* = 84) had been included in a multi-center study on de novo patients analyzing quality of life and pain after onset of BoNT/A-therapy [14].

### Statistical analysis

**Fig. 1:**
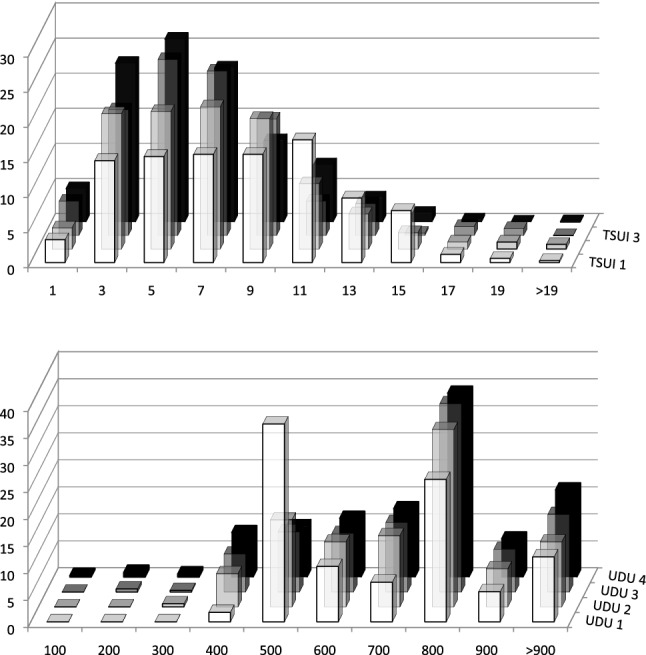
Upper part: distributions of the relative frequencies of the TSUI-scores before the first (TSUI 1), the second (TSUI 2), the third (TSUI 3), and the fourth (TSUI 4) injection in 337 de novo patients with idiopathic CD (injected during the last 12 years). All four distributions are significantly different: the initial distribution is quite broad, the distributions become smaller with time, and a peak develops around a TSUI-score of 4–5 with a duration of therapy. Lower part: distributions of the relative frequencies of the BoNT/A-doses (uDU 1, uDU 2, uDU 3, and uDU4) of the first four injections. The peak around 500 uDU (which is prominent in the dose distribution of the first injection) decreases with time. Higher doses were used more frequently for injections 2–4

**Fig. 2:**
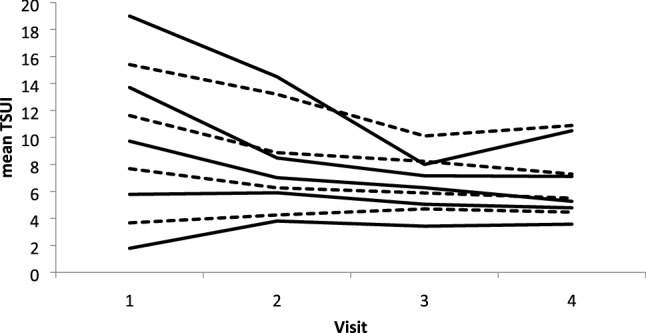
For all four visits, mean TSUI-scores for the TSUI-ranges (see also “Methods” section) presented in Fig. 1 were calculated. The lines tend to converge with ongoing BoNT/A-therapy, but in principle, the order of the lines remains preserved with the increasing number of injections. It is quite obvious that patients with an initial high score improve, whereas severity of CD in patients with a low initial TSUI-score does not change or even worsens

For data presentation and calculation of distributions, patients were grouped into the following different TSUI-ranges (0–1, 2–3, …, 18–19, > 19; comp. Fig. 1 upper part) and unified dose ranges (1–100, 101–200, …, 801–900, > 900; comp. Fig. 1 lower part). The relative percentages of the number of patients in these TSUI- or dose ranges are presented in Fig. 1, so that the sum of all relative percentages is 100. For all the TSUI-ranges, TSUI-mean values were calculated for all four visits (Fig. 2). Improvement after injection 1 was determined as the difference between the TSUI-score at visit 2 (TSUI 2) minus TSUI-score at visit 1 (TSUI 1). Improvement after injection 2 (TSUI 3–TSUI 1) and 3 (TSUI 4–TSUI 1) was determined similarly.

**Fig. 3:**
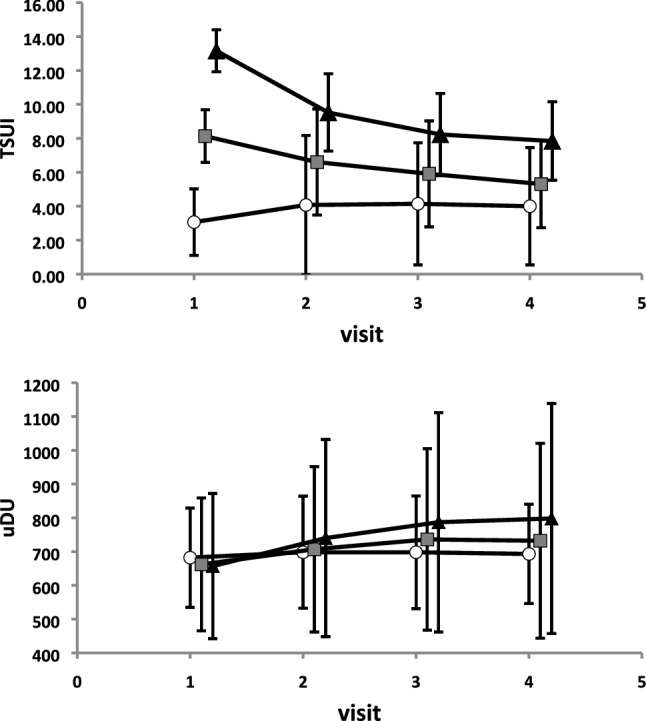
Upper part: mean TSUI-scores of the HIS-subgroup (initial TSUI-score > 10; black triangles) significantly decrease with duration of treatment. In the MIS-subgroup (initial TSUI-score 6–10; gray squares), mean TSUI-scores also improve with time. However, in the LIS-subgroup (initial TSUI-score < 6; open circles), TSUI-scores worsen with time. Lower part: mean unified doses in the HIS-subgroup were slightly increased with duration of treatment. In the MIS-subgroup, mean unified doses were also increased. In the LIS-subgroup, unified doses were not changed

**Fig. 4:**
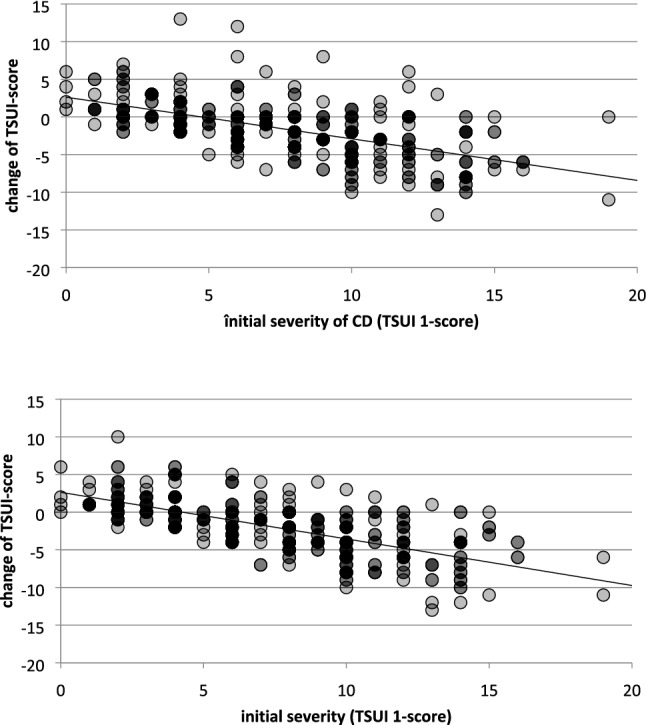
Upper part: on the ordinate, the differences between the severity 3 months after the first injection (TSUI 2) and the severity before the first injection (TSUI 1) are plotted against the baseline TSUI-score (TSUI 1; abszissa) for all patients. Positive values indicate worsening 3 months after the first injection, negative values indicate improvement. (Points are plotted with transparency. Therefore, darkness of a point depends on the number of patients with the same *x*, *y*-values.) Heavy lines indicate the range of improvement. Worsening is found more in patients with a low initial TSUI-score. Lower part: on the ordinate, the difference between the severity 3 months after the third injection (TSUI 4) and the severity before the first injection (TSUI 1) is plotted against the baseline TSUI-score (abszissa). Positive values indicate worsening 9 months after the onset of BoNT-therapy 3 months after the third injection, and negative values indicate improvement. Compared to the improvement after the first injection (left side) more improvement is seen after the third injection, the regression line has a steeper slope

Furthermore, for statistical comparisons (Fig. 3), three patient-subgroups were distinguished: the LIS-group (*n* = 111: patients with a **l**ow **i**nitial TSUI-**s**core < 6), the MIS-group (*n* = 149: patients with a **m**oderate **i**nitial TSUI-**s**core between 6 and 10), and the HIS-group (*n* = 77: patients with a **h**igh **i**nitial TSUI-**s**core > 10). Between subgroup differences were tested by a repeated-measure two-way ANOVA, detailed differences were tested by means of a posthoc analysis (Tukey’s HSD-test). Differences between TSUI-score and dose distributions were analyzed by means of a Friedman-test. For correlation analysis, the Pearson product–moment correlation was used (see Fig. 4). All statistical procedures and tests were part of the SPSS statistics package (SPSS, version 25, IBM, Armonk, USA).

## Results

### Demographical data

Demographical data of the 337 CD-patients whose data were used for the final analysis were typical for a cohort of patients being referred to receive BoNT/A-therapy (comp. [11]). The ratio between female and male patients was 1.72, and the mean age at onset of BoNT/A-therapy was 48 ± 9.2 years (age range: 18–73 years).

### Distributions of TSUI-scores and BoNT doses in our cohort of de novo CD-patients

The TSUI-score (possible range from 0 to 25) at the first visit (just before the first BoNT/A-injection) ranged from 0 to 22 [mean TSUI: 7.61 ± 4.05; comp. Fig. 1 (upper part)]. In 111 patients, the initial TSUI-score was smaller than 6 (LIS-subgroup); in 149, the initial Tsui-score ranged between 6 and 10 (MIS-subgroup); and in 77 patients, the TSUI-score was larger than 10 (HIS-subgroup). Mean ages of these three patient-subgroups did not differ. No correlation between age at onset of therapy and initial severity of CD could be detected neither in the entire cohort nor in these three subgroups. The distribution of initial TSUI-scores was broad with a small peak around 10–11 [Fig. 1 (upper part)]. With ongoing therapy, the distribution of the TSUI-scores became smaller with a progressively pronounced peak around 4–5.

The distribution of the unified doses [Fig. 1 (lower part)] used for the first injection was bimodal with a clear-cut peak around 500 uDU and a second peak around 750 uDU. With ongoing therapy in spite of a significant improvement, doses were increased, so that the peak around 750 uDU further increased and the peak around 500 uDU disappeared. Statistical testing of the distributions (Friedman-tests) showed that all four TSUI- and all four dose distributions were significantly (*p* < 0.05) different.

### Different outcome in CD-patients with different initial severity

For 9 of the 11 TSUI-ranges [used for the distribution in Fig. 1 (upper part)], enough data were available to calculate TSUI-mean values for all four visits (Fig. 2). It is quite striking that the lines corresponding to high initial TSUI-score ranges decrease with ongoing BoNT/A-therapy and that the lines corresponding to low initial TSUI-score ranges increase with ongoing therapy. Apart from a few minor exceptions, the lines tend to converge with the number of injections applied to the patients, but the order among the lines is preserved in principle (Fig. 2).

For further statistical comparisons, mean TSUI-scores [Fig. 3 (upper part)] and mean doses [Fig. 3 (lower part)] were calculated for the LIS- (open circles), the MIS- (gray squares), and the HIS-subgroup (black triangles). The TSUI-scores of all three subgroups were significantly different for all four visits (*p* < 0.001; Tukey’s HSD-test), the unified doses did not differ for all four visits (n.s., Tukey’s HSD-test). In spite of the use of comparable (n.s.) mean doses in all three subgroups, the TSUI-scores of the HIS- and MIS-subgroups highly significantly (*p* < 0.001) improved, whereas the TSUI-scores in the LIS-group significantly worsened [*p* < 0.001; Fig. 3 (upper part)]. The relative improvement after 3 injections [(TSUI1-TSUI 4)/TSUI 1 × 100 = 44%] was significantly larger (*p* < 0.01) in the HIS-subgroup compared to the MIS-subgroup (35%). In the LIS-subgroup, the ratio TSUI 4/TSUI 1 × 100 significantly (*p* < 0.001) increased from 100 to 180% corresponding to a worsening of up to 80%. In both the HIS- and MIS-subgroup, mean BoNT/A-doses for injection 2–4 were slightly (n.s.) increased; in the LIS-subgroup, BoNT/A-doses were kept constant [Fig. 3 (lower part)]. At visits 2, 3, and 4, unified doses and changes of dose did not differ between all three subgroups because of the large interindividual variation.

### Improvement rates in the LIS-, MIS-, and HIS-subgroup

**Fig. 5:**
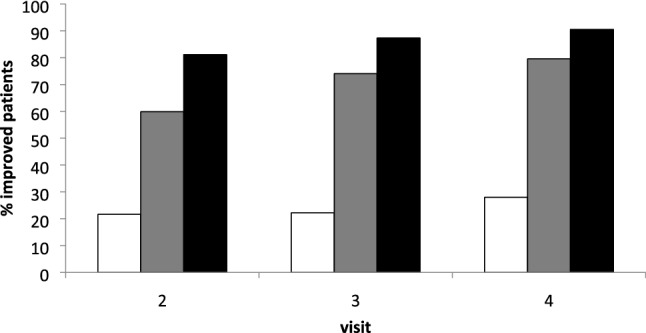
When the percentage of patients is determined who respond to the first, second, and third injection in the LIS- (open), MIS- (gray), and the HIS- (black) subgroup (by analyzing in how many patients, the differences TSUI2-TSUI1 (visit 2), TSUI3-TSUI1 (visit 3), and TSUI4-TSUI1 (visit 4) are negative (= improvement), it is quite obvious that in the HIS-subgroup, most patients respond to the first injection, less in the MIS-subgroup and less than 25% in the LIS-subgroup. There is a further increase in the percentage of responding patients in the HIS- and MIS-subgroup with the next two injections, but only a little further increase in the percentage of improvement in the LIS-subgroup

To analyze in more detail the different response behavior to BoNT/A-therapy in the three subgroups, the differences TSUI 2–TSUI 1, TSUI 3–TSUI 1, and TSUI 4–TSUI 1 (see “Methods” section) were calculated for each patient. The percentage of patients with a negative difference (= improvement) is presented in Fig. 5. In the HIS-subgroup (black bars), an improvement is observed in more than 80% of the patients already after the first injection. With further injections, improvement rate increases up to more than 90%. In the MIS-subgroup (gray bars), significantly fewer patients (*p* < 0.05) improved after the first injection. However, with the next two injections, the improvement rate increases to more than 80%. In the LIS-subgroup, improvement rate remains less than 30% even after the third injection.

### Comparison of improvement and worsening on an individual basis

The large error bars in Fig. 3 demonstrate that considerable interindividual variability in the response behavior is present in the entire cohort. Therefore, to quantify the individual outcome, the differences TSUI 2–TSUI 1, TSUI 3–TSUI 1, and TSUI 4–TSUI 1 were determined for all patients and plotted against the baseline score (TSUI 1; abszissa). A positive difference indicates worsening, a negative difference improvement 10–14 weeks after the 1st, 2nd, and 3rd injection, respectively. In Fig. 4, the outcomes after the first (upper part) and the third injection (lower part) are presented. In both parts of Fig. 4, it is obvious that some patients with a low initial TSUI-score (< 6) tend to worsen much more than patients with a high initial TSUI-score (> 10). The regression line between outcome and initial TSUI-score becomes increasingly steeper (comp. Fig. 4 upper and lower parts), but the intercept on the ordinate remains nearly constant. This is due to a progressive improvement in the more severely affected patients but nearly no change or even worsening in the mildly affected patients.

## Discussion

### Distribution of initial severity of CD in the present cohort of de-novo CD-patients

The presented cohort of 337 botulinum toxin naïve patients suffering from idiopathic CD includes nearly all de novo CD-patients having presented in our BoNT-outpatient clinic during the last 12 years. On average, we see 25–30 de novo CD-patients per year. Compared to previously described cohorts of CD-patients being referred to our department [8, 14, 15] or to other centers in Germany [11] to receive botulinum toxin injections for the first time, mean age at therapy onset was 48 ± 9.2 years and rather low. In the multi-center study across Germany and Austria testing a treatment algorithm for de novo CD-patients, mean age at onset of therapy was 51.9 ± 12.7 years for all 515 patients. This difference in mean age at onset of therapy results from an earlier referral to BoNT/A-therapy compared to previous years. As a consequence of early referral mean, TSUI-score was lower (7.61) and the variability of the severity of CD (SD/TSUI × 100 = 4.05/7.61 × 100 = 53.2%) higher compared to previously described cohorts (8,14,15; for example, the mean TSUI-score was 8.4 and SD/TSUI × 100 = 3.5/8.4 × 100 = 41.7% in [11]). About one third (111/337) of the cohort had an initial TSUI-score < 6. This LIS-subgroup was large enough for a statistical comparison with the more severely affected patients (MIS- and HIS-subgroups).

### Distribution of the severity of CD reveals a systematic treatment effect

Comparisons of the TSUI-score distributions for visit 1–4 (Fig. 1) clearly demonstrate that the broad spectrum of initial TSUI-scores is altered by a systematic treatment effect. With treatment duration, TSUI-scores converge from above and from below (Fig. 2) and tend to cluster around a value around 4–5 (Figs. 1 and 2). This is in full agreement with another study on very long-term treatment in our BoNT-outpatient clinic showing that in the mean the TSUI-scores ly between 4 and 5 regardless of whether patients were continuously treated for only 2 or up to 22 years [16]. Changes of the severity of CD go along with changes of quality of life. This can be observed not only after a single injection, but also after long-term treatment over 22 years [17, 18]. Interestingly, the results of the CDQ24 questionnaire asking for 24 aspects of quality of life of CD-patients were better after the first BoNT/A-injection at week 12 than after week 4 [17]. Patients and physicians assessed the effect of the first BoNT/A-injection at week 12 at least as good as at week 4. The correlation between CDQ24 and TSUI-score even tended to be better at week 12 than at week 4 [17].

Because of this correlation between QoL and CD-severity, patients in the LIS-group are at risk to cessate BoNT/A-therapy. They do not experience an improvement of severity of CD and therewith of quality of life.

### Is there evidence for a different outcome in special patient-subgroups of CD?

Apparent reasons for reduced efficacy of BoNT/A-therapy in de novo patients with idiopathic CD are contractures, degenerative diseases of the cervical spine, radiculopathies, and associated pain syndromes [9, 10, 19, 20]. Secondary treatment failure may occur early in the course of treatment [14], but, usually, it takes years until relevant titers of neutralizing antibodies are induced [21, 22]. Otherwise, no apparent reasons seem to exist reducing the efficacy of BoNT/A-therapy. Responder rates usually well above 50% with a reduction of severity of CD up to 60% with repeated injections (for an overview, see Refs. [4, 9]). The percentage of primary non-responders is reported to be less than 4% [4]. Patients with torticollis and laterocollis respond to injections with 500 MU abo-BoNT/A equally well [11]. Even in depressed patients, the clinical effect rated by the treating physician is equal to that of non-depressed patients. However, depressed patients seem to assess the benefit of BoNT/A-therapy less compared to non-depressed patients [17].

Furthermore, it has been reported that relative improvement (improvement divided by initial severity) is similar for all patients irrespective of the severity and complexity of CD [8]. Obviously, this statement is at variance with the results of the present study clearly demonstrating a dependence of relative improvement on the initial severity of CD in all figures, especially in Figs. 3 and 4). The reason for this discrepancy results from the fact that Kessler et al. [8] have only analyzed moderately and severely affected patients and have extended their observations also to CD-patients with low initial severity. Thus their results are in full agreement with the present study but not their conclusions.

### Are the current results a trivial consequence of too low sensitivity of the TSUI-score?

In the upper TSUI-score ranges, the scale is more sensitive to detect subtle relative improvement. Patients with a TSUI-score of zero cannot be improved any further. Therefore, it is entirely reasonable to hypothesize that with lower initial TSUI-scores absolute and relative improvement after a BoNT/A-injection will decrease. However, a significant worsening was observed in patients with low initial severity. That cannot be explained by too low sensitivity of the TSUI-score to detect an improvement in the low score range. From the literature, one would even have expected that “patients with simple cervical dystonia, such as rotation or tilt, and with shorter duration of symptoms usually respond better” [4, 8]. If this was true, too low sensitivity of the TSUI-scale would have led to a small or no change of severity, but definitely not to a significant worsening.

### Explanation of the results by an underlying disease progression

We, therefore, prefer the explanation that most of the de novo CD-patients being referred to BoNT/A-therapy with low initial severity of CD do not have reached a stable plateau of their disease severity [4]. Therefore, these patients may deteriorate in spite of BoNT/A treatment.

We do not think that all these botulinum toxin naïve CD-patients worsening after the first 1–3 BoNT-injections are all primary non-responders. These patients usually have a clear clinical effect with a clear peak effect 2–4 weeks after a BoNT-injection, followed by a decline of this effect thereafter [4, 7]. However, we think that the disease of the central nervous system (CNS) underlying CD still progresses even after the onset of BoNT/A-therapy in these de novo patients, so that severity of CD (scored 10–14 weeks after a previous injection during the declining phase of the clinical effect) even becomes worse compared to baseline severity. These results are in full agreement with the hypothesis that BoNT/A-mediated reduction of muscle tone and strength is a symptomatic treatment, but not a disease-modifying therapy of CD.

### Implications for patient’s content, patient management, and future scientific studies

Since it is likely that de novo CD-patients with a low initial severity may deteriorate in spite of BoNT-injections with sufficiently high doses of BoNT, these patients should be informed carefully about the realistic goal of treatment (see “Distribution of the severity of CD reveals a systematic treatment effect” of the “Discussion” section). Otherwise, they will not be content with BoNT-therapy, will be disappointed, and stop BoNT-therapy. Several patients (not included in the present study with a low severity at the onset of therapy) have been referred to our center as primary non-responders who had cessated BoNT/A-therapy after the first few injections, responded well in our center after a further deterioration following cessation of BoNT/A-therapy had occurred.

For scientific studies, the present data may have the implication that not only mean severity has to be kept balanced between different arms of treatment but also the proportion of mildly and more severely affected patients. Otherwise, a smaller response may result in the arm of the study with a higher proportion of mildly affected patients. In most of the studies on BoNT-treatment of cervical dystonia, patients with a low initial severity have been excluded from the very beginning. This is probably the reason why the influence of initial severity has not been studied in detail so far.

Because of the relevance of the present observations, it is proposed to perform multicentric and prospective studies comparing response behavior in mildly and severely affected CD-patients and analyzing the influence of the duration of the disease before the onset of BoNT-therapy in more detail.

## Availability of data and material

The data that support the findings of this study are available from the corresponding author, Prof. Harald Hefter, upon reasonable request.

## Data Availability

SPSS version 24 and ACCESS data bank of the research center of university hospital Duesseldorf.
